# Behavioral Impairment and Amnesia at the Onset of Susac Syndrome

**DOI:** 10.7759/cureus.38089

**Published:** 2023-04-25

**Authors:** Marc Masjuan, Trajche Ivanovski, Helena Sarasibar Ezcurra, Elena Rigo Oliver

**Affiliations:** 1 Neurology, Hospital Universitario Son Llatzer, Palma de Mallorca, ESP; 2 Radiology, Hospital Universitario Son Llatzer, Palma de Mallorca, ESP; 3 Ophthalmology, Hospital Universitario Son Llatzer, Palma de Mallorca, ESP

**Keywords:** immunosuppressive treatment, corpus callosum lesions, sensorineural hearing loss, branch retinal artery occlusion, encephalopathy, autoimmune microangiopathy, susac syndrome

## Abstract

Susac syndrome (SS) is an autoimmune microangiopathy that affects the brain, retina, and inner ear, causing a wide range of clinical manifestations. The triad of encephalopathy, visual disturbances, and hearing loss constitute the classic disease presentation.

We describe an original clinical case of a young male with a definitive diagnosis of SS, who presented with disordered behavior and amnesia, initially manifested as a dissociative or anxiety disorder but with a fulminant evolution toward severe encephalopathy associated with retinal infarcts and sensorineural hearing loss.

After the diagnosis of SS, aggressive immunosuppressive treatment was started with significant neurological improvement and favorable evolution during the follow-up period. SS is a rare but potentially devastating disease that can cause great disability if not properly diagnosed and treated. The onset of SS with behavioral or psychiatric manifestation can be misleading, causing a diagnostic delay.

## Introduction

Susac syndrome (SS) is a rare, immune-mediated microangiopathy that affects the small blood vessels of the brain, retina, and inner ear. The classic triad of encephalopathy, visual impairment, and hearing loss or tinnitus raises the alarm of SS, although it is uncommon at disease onset or can be easily overlooked if encephalopathy predominates.

The purpose of this case is to raise awareness of SS as a potential cause of subacute behavioral or psychiatric disorders in young adults. We also emphasize the need for prompt treatment with immunosuppressive therapy to achieve a favorable outcome and prevent disability.

## Case presentation

A young male in his 20s with a history of cluster B personality disorder, heavy cannabis consumption, and sporadic amphetamine and ecstasy use attended the emergency department of our hospital due to behavioral changes and memory failure in the last week. According to his mother, he was extremely nervous, irritable, and distracted; he had not gone to work recently and he could not remember where he had parked the car.

The patient was inattentive and partially disoriented with time. There were no focal signs on examination and a head CT and blood tests were unremarkable. So after the initial assessment, the presumptive diagnosis was a dissociative or anxiety disorder triggered by abuse of toxic substances. Treatment with anxiolytics was started, and he was referred to a psychiatric outpatient clinic for their expert opinion.

However, he was brought back two weeks later because of significant worsening with decreased spontaneous language, inappropriate behavior, difficulty in walking, and urinary incontinence. On arrival, he was afebrile with normal vital signs. Neurological examination revealed severe apathy and akinetic mutism. The patient was unable to stand or walk independently. No oculomotor disorder, cranial nerve deficit, or muscle weakness was observed. The musculoskeletal reflexes were normal, and the plantar cutaneous reflexes were flexor. He had no neck rigidity or meningeal signs. The rest of the clinical examination was unremarkable.

**Figure 1:**
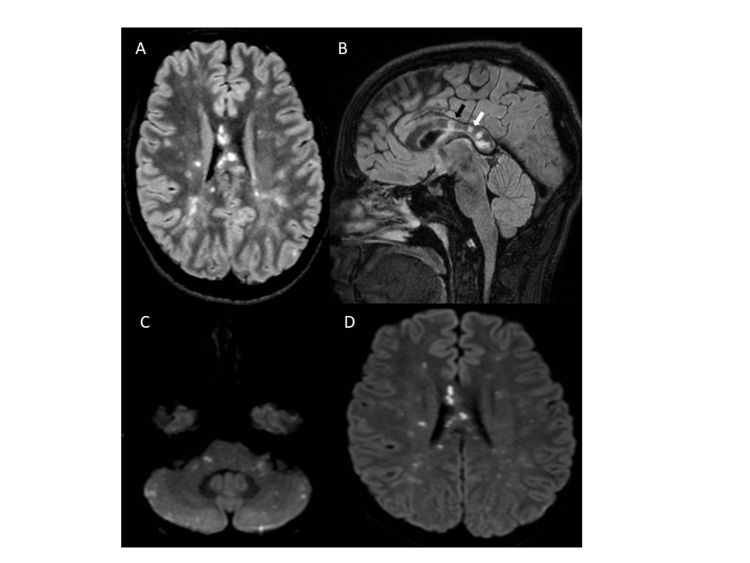
Cerebral MRI images on admission (A-D) (A) Periventricular, subcortical and callosal lesions in the axial FLAIR section. (B) Characteristic snowball (white arrow) and “icicle” (black arrow) lesions in the corpus callosum. C and D. Supratentorial and infratentorial small acute ischemic lesions on DWI sequence.

The second head CT performed in the emergency department was normal. Cerebrospinal fluid (CSF) analysis revealed a lymphocytic pleocytosis (14 leucocytes/mm^3^) and elevated protein (228 mg/dL). Oligoclonal bands and antineuronal antibodies were absent. PCR tests for neurotropic viruses were negative. Cerebral MRI demonstrated multiple supratentorial and infratentorial cortical, subcortical, and periventricular small T2-hyperintense lesions, many with restricted diffusion and no gadolinium enhancement. Multiple central lesions throughout the corpus callosum (snowball lesions) could be observed (Figures 1A-1D).

Electroencephalography showed diffuse slowing without epileptiform discharges. Toxicological analysis of the urine was positive only for cannabis. Other routine laboratory analyses, including thyroid hormones and angiotensin-converting enzymes, were unremarkable. Antinuclear antibodies, anti-neutrophil cytoplasmic antibodies, and antibodies related to antiphospholipid syndrome (APS) were negative.

**Figure 2:**
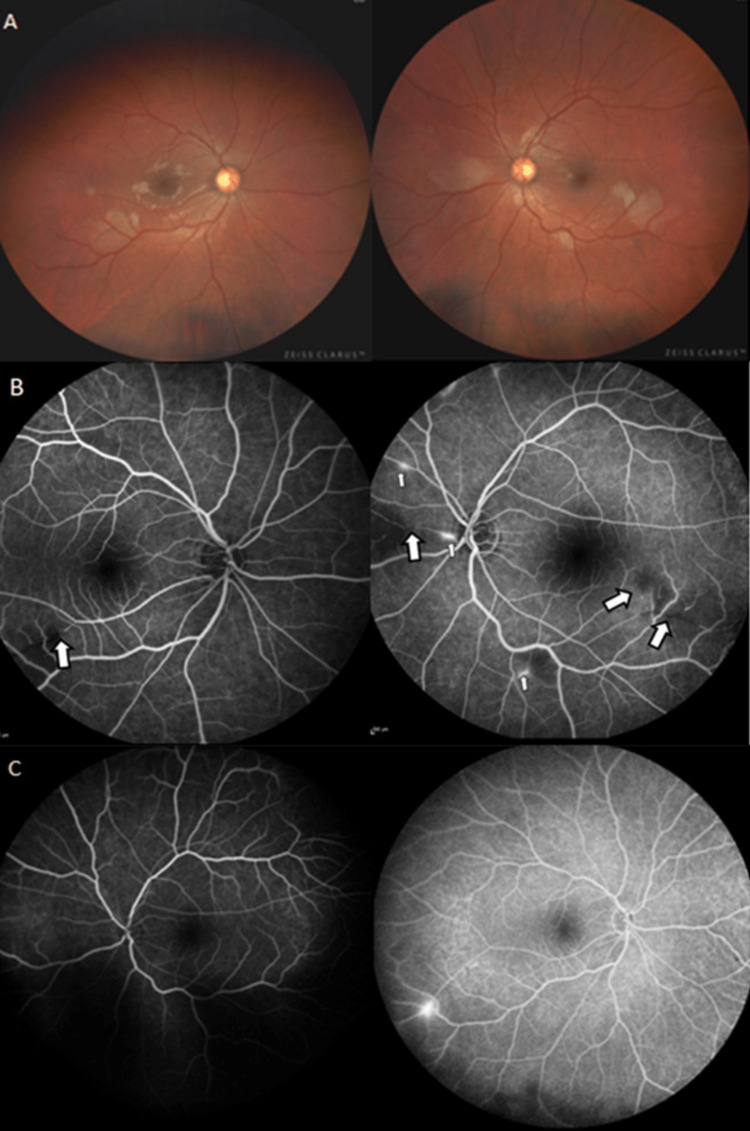
Wide-field retinography and retinal fluorescein angiography findings in both eyes before and after treatment (A-C) (A) Areas of retinal whitening in both eyes. (B) Fluorescein angiography: characteristic branch retinal artery occlusion (thick arrows) with arteriolar wall hyperfluorescence and leakage (thin arrow). (C) Control fluorescein angiography three months later: almost complete recovery of the artery occlusions and arterial wall hyperfluorescence.

Eye fundus examination showed areas of retinal pallor in both eyes compatible with areas of branch retinal artery occlusion (BRAO) (Figure 2A). Retinal fluorescein angiography (RFA) confirmed this finding, also showing hyperfluorescence and leaking of fluorescent dye (Figure 2B) at the areas of occlusion.

**Figure 3:**
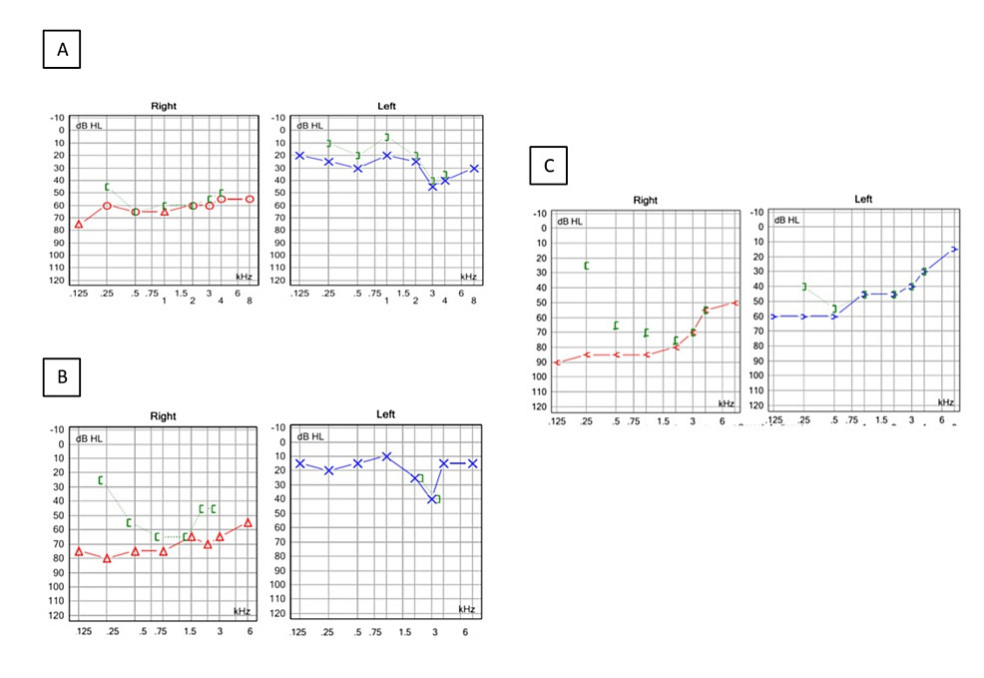
Tone audiometry (A-C) (A) (at onset) Bilateral sensorineural hearing loss. In the right ear is pantonal, predominant low-to-middle frequencies and high frequencies hearing loss in the left ear. (B) (one month later) Without significant changes after initial treatment. (C) (five months later) New left-side hearing loss, no significant change on the right side.

Pure tone audiometry revealed bilateral, right predominant low-to-mid frequency sensorineural hearing loss (SNHL) (Figures 3A-3C).

Treatment was started with intravenous (i.v.) methylprednisolone 1 g/day for five days followed by an oral prednisone maintenance dose of 1 mg/kg/day for 10 days, with only mild improvement in his mental status and language. During this period, he initiated aggressive behavior that required the administration of haloperidol, quetiapine, and olanzapine. The recovery was not satisfactory at this point, so we added immunoglobulins i.v. (0.4 g/kg/day) for five consecutive days and simultaneously cyclophosphamide 900 mg i.v. two administrations within a 14-day interval, with significant clinical improvement. We repeated the immunoglobulins (0.5 g/kg/day for two consecutive days) every three weeks and started tapering prednisone. Mycophenolate mofetil acid was added at discharge, five weeks after admission and the dosage increased to 1,000 mg twice per day. One week after discharge, intratympanic dexamethasone was administered due to the absence of significant improvement in hearing loss.

**Figure 4:**
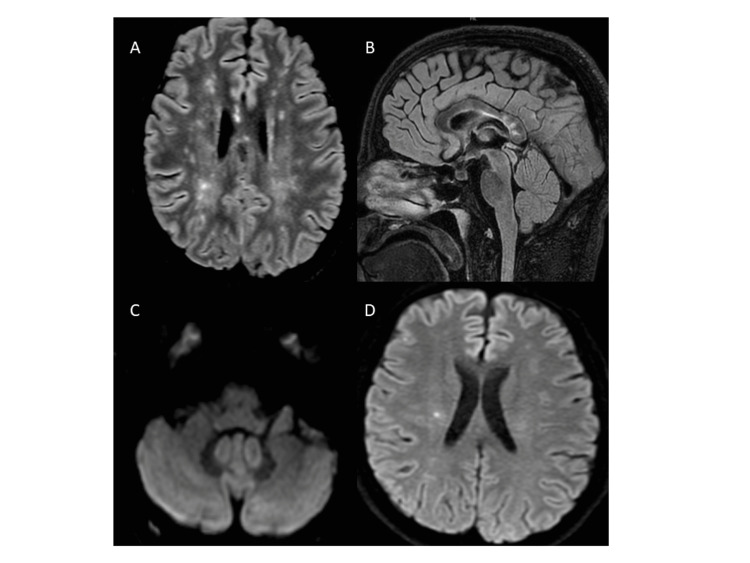
Cerebral MRI images three months after discharge (A-D) (A, B) Reduction in the number and size of the lesions on axial and sagittal FLAIR sections. (C, D) Only a few punctate lesions can be observed on DWI sequence.

After one month of treatment, the patient was tranquil and attentive; he was speaking fluently and could walk independently with only mild gait ataxia. Three months after discharge, he was functionally independent with mild impairment of attention, processing speed and verbal fluency as well as mild alteration of working memory and visuoconstructive apraxia. However, he still had not recovered from the right-side hearing loss despite intratympanic doses of dexamethasone. On follow-up after three months, the brain MRI (Figures 4A-4D) showed a decrease in the number and size of existing lesions, without the appearance of new lesions.

The RFA showed almost complete recovery of the ischemic lesions (Figure 2C). Five months after the disease onset, he was readmitted for abrupt left-side sensorineural loss (Figure 3C). Rituximab i.v. 1,000 mg was administered twice within a two-week interval. Eight months after the exacerbation; there was no improvement in the sensorineural loss.

## Discussion

SS is a rare, autoimmune-meditated microangiopathy that causes microinfarcts that affect the brain, retina, and inner ear, leading to a wide range of clinical manifestations. The triad of encephalopathy, visual disturbances with BRAO and SNHL is the classic manifestation of the disease [1]. The annual incidence of SS is estimated to be in the range of 0.024-0.13 per 100,000 [2,3], with 60% of individuals aged between 21 and 35 years at the time of diagnosis and a reported male:female ratio of 1:3.5 [1]. The clinical course in SS may be monocyclic, polycyclic, or chronic continuous.

The syndrome shares histological similarities with juvenile dermatomyositis, such as adventitial thickening and endothelial cell swelling in precapillary arterioles, sometimes to the point of luminal occlusion and perivascular lymphocytic infiltration [4]. Considering the lack of solid clinical experience with the disease, a similar treatment approach is being used in the case of SS [5]. The most common presentation at the onset is encephalopathy, characterized by cognitive impairment, mainly memory, attention, and executive dysfunction or confusion and disorientation. Migraine-like headaches might be present at disease onset as well [1]. The visual and hearing alterations usually present in the following weeks after the encephalopathy but can also be the initial or unique clinical manifestation. It is important to outline that diagnosis can be challenging when the clinical triad is not present at disease onset [6].

In our case, the preceding personality disorder and heavy drug abuse were misleading and led to the equivocal assumption that the initial symptoms were caused by a psychiatric disorder. Similar cases have already been reported in the literature [7,8]. Although SS has been described in patients with a history of drug abuse, there is no substantial evidence that toxins can trigger SS. The diagnosis is clinical-radiological and is definitive when all components of the triad can be demonstrated [9,10].

Involvement of the corpus callosum is characteristic and highly suggestive. It usually consists of small, hyperintense lesions on T2-weighted/FLAIR sequences affecting the central part of the corpus callosum (snowball) extending to the roof of the corpus callosum (Figure 1B), unlike demyelinating lesions, which typically affect its floor and ventricular interface. When acute, these lesions can show signal changes in DWI imaging (Figures 1C, 1D). Other MRI findings include periventricular and subcortical white matter lesions and brainstem and cerebellar lesions; in the chronic phase, a residual “black hole” on sagittal T1-weighted images might be seen [11].

We wish to remark on the difficulty of assessing visual or auditory symptoms in patients with encephalopathy. Our patient only reported vision and hearing problems once the encephalopathy had significantly improved. In the case of possible SS (central nervous involvement), it is recommended to look for specific signs such as BRAO and arterial wall hyper fluorescence (AWH) in RFA as well as SNHL on audiometry. AWH has not been observed in other types of vasculitis affecting the retina and could therefore be considered pathognomonic of SS (Figure 2C) [12]. All patients should be evaluated with audiometry. NHL commonly affects the low and middle frequencies and is often irreversible [13].

The treatment of SS is as challenging as the diagnosis. Treatment recommendations for SS are empiric and based on the experience with juvenile dermatomyositis as mentioned before [14]. There are no randomized or controlled clinical trials due to the rarity of the disease. Our treatment decisions are based on the recommendations from one set of guidelines [12] as well as on previous case series [8,15-17]. A common initial therapy is corticosteroids followed by immunoglobulins and additional immunosuppressive drugs like cyclophosphamide or rituximab. In some cases, aspirin might be an option [15].

Intratympanic injection of dexamethasone in the acute phase of hearing loss and tinnitus may provide a benefit. In patients with profound SNHL, cochlear implants may be the best option [18]. It is important to emphasize that our patient was able to make a considerable cognitive recovery despite significant encephalopathy at presentation. This does not always come across in the literature [19]. Failure to use aggressive immunosuppression from the outset often leads to ongoing relapses which can be detrimental to the patient.

## Conclusions

SS is a rare but potentially devastating disease that can cause great disability if not properly diagnosed and treated. The onset of SS with behavioral or psychiatric manifestations can be misleading, causing a diagnostic delay. The MRI and RFA findings are highly suggestive and crucial for the diagnosis. Prompt diagnosis and aggressive immunosuppressive treatment are needed in order to avoid irreversible damage and achieve a better outcome.
